# Associations of static anatomical geometry with planned dosimetric endpoints in prostate radiotherapy: a multicenter study

**DOI:** 10.3389/fonc.2026.1809765

**Published:** 2026-07-06

**Authors:** Weixiang Lin, Yongwen Fang, Ying Chen, Chongmin Liang, Zhanwei Li, Xiaosheng Lin, Jianlan Fang, Junwei Chen, Liangjie Xiao

**Affiliations:** 1 Department of Radiation Oncology, Ganzhou Cancer Hospital, Ganzhou, China; 2 State Key Laboratory of Oncology in South China, Guangdong Provincial Clinical Research Center for Cancer, Sun Yat-sen University Cancer Center, Guangzhou, China

**Keywords:** anatomical geometry, dosimetry, DVH, multicenter, prostate radiotherapy, quality control

## Abstract

**Purpose:**

To investigate associations between static anatomical geometry and planned dosimetric endpoints in prostate radiotherapy using a unified multicenter workflow, with particular emphasis on OAR dose metrics.

**Methods:**

We analyzed 37 patients from four centers with planning CT and DICOM-RT objects (RTSTRUCT/RTPLAN/RTDOSE). ROI masks were rasterized from RTSTRUCT. Dose grids were reconstructed from RTDOSE, resampled to the CT grid for voxel-wise dose sampling, and DVH metrics were recomputed using a unified implementation. We extracted geometric features of targets and organs at risk (OARs), including volume and longitudinal extent, and evaluated their associations with key planned dosimetric endpoints, with particular emphasis on OAR dose metrics. Target coverage metrics were analyzed as secondary exploratory endpoints. A systematic QC audit was performed for atypical dose results. In addition, an independent institutional DICOM cohort was used to assess workflow applicability in a real-world clinical setting.

**Results:**

In the main cohort, bladder volume and longitudinal extent showed consistent, directionally aligned associations with multiple bladder DVH endpoints. Prescription-normalized sensitivity analyses in cases with explicit RTPLAN target prescription-dose information showed similar bladder-related patterns. Near-zero bladder D_2cc_ values were interpreted as reflecting geometric separation from the high-dose region rather than evident errors in dose reconstruction or DVH computation. The same processing workflow was successfully applied to the independent institutional DICOM cohort.

**Conclusion:**

Under contemporary planning and PTV-margin practices, inter-patient anatomical geometry was consistently associated with planned OAR dosimetric endpoints within a static planned-dose (RTDOSE-based) DVH evaluation.

## 1 Introduction

External beam radiotherapy (EBRT) is a cornerstone treatment for localized and locally advanced prostate cancer (1). The widespread adoption of IMRT/VMAT and image-guided radiotherapy (IGRT) has improved dose conformality while enabling more stringent sparing of organs at risk (OARs) (2). However, steeper dose gradients also increase sensitivity to geometric uncertainties. Consequently, even small anatomical variations or setup deviations may translate into meaningful differences in the planned dose distribution and dose–volume histogram (DVH) metrics, potentially compromising target coverage and increasing high-dose exposure to OARs (3, 4).

Internal anatomical variation is a major source of geometric uncertainty in prostate radiotherapy (5). Changes in bladder filling, rectal contents (e.g., gas migration), and pelvic soft-tissue deformation can shift and deform the prostate and seminal vesicles relative to bony anatomy, thereby altering the spatial relationship between targets and adjacent OARs (bladder and rectum) (6). These effects are most pronounced in plans with steep dose gradients, where millimeter-scale geometric changes can yield measurable dosimetric consequences. Such consequences commonly manifest as reduced near-minimum target-dose metrics (D_95_, D_98_, D_min_) and/or increased OAR dose metrics (D_mean_, D_2cc_) (7, 8). Although bladder and rectal preparation is routinely used to mitigate variability, residual inter-patient and interfraction anatomical changes remain frequent and cannot be fully eliminated (8).

Prior work has primarily focused on bladder/rectum dosimetry and toxicity risk, whereas target (PTV/CTV) dose-coverage stability is often treated as an assumed property rather than a primary endpoint (9). In addition, internal anatomy is frequently summarized by volume or a single distance metric, while fewer studies have systematically incorporated reproducible geometric descriptors—such as longitudinal physical extent—that can be computed consistently across centers and directly linked to key target DVH endpoints. These limitations are further amplified in multicenter settings: inconsistent ROI semantics can hinder harmonization and reduce cross-center comparability (10), and DVH generation and reporting may vary systematically across commercial dosimetry systems (11), complicating cross-study and cross-center interpretation.

To address these gaps, we analyzed a publicly available multicenter prostate radiotherapy dataset and implemented a unified processing and quality-control pipeline. All cases were handled under harmonized ROI semantics within a shared DICOM physical coordinate system. We generated 3D structure masks and recomputed static DVH metrics directly from RTDOSE using a standardized implementation (12). Our primary objective was to evaluate associations between reproducible geometric features and planned dosimetric endpoints within a unified multicenter workflow, with particular emphasis on OAR dose metrics. Target coverage metrics were retained as secondary exploratory endpoints to assess whether any residual geometry-related variation remained detectable despite contemporary plan normalization and margin-based design. Together, these analyses help clarify how reproducible static anatomical geometry is associated with planned dosimetric endpoints in contemporary prostate radiotherapy. Clinically, such geometry-derived information may be most useful as an early planning-support signal rather than a stand-alone decision tool. Features available directly from the planning CT/RTSTRUCT could help flag cases that warrant closer review of OAR sparing, stricter bladder/rectal preparation, or consideration of re-simulation/re-planning when the anatomy appears unfavorable for routine planning trade-offs.

## 2 Materials and methods

### 2.1 Data sources and case selection

Two data sources were included: a publicly available multicenter dataset and routine clinical data from Ganzhou Cancer Hospital. The public dataset was obtained from the open-access resources of the TROG 15.01 SPARK clinical trial (NCT02397317) and comprises prostate radiotherapy planning DICOM-RT objects (planning CT, RTSTRUCT, RTPLAN, and RTDOSE), together with supporting information for intrafraction motion monitoring. Intrafraction motion data were derived from KIM-based tracking in the SPARK dataset. The public multicenter cohort was therefore analyzed in the context of an image-guided radiotherapy (IGRT)-enabled workflow, with KIM serving as the image-guidance modality for intrafraction target-motion monitoring. These motion records were interpreted as target-related intrafraction motion monitoring and did not directly quantify bladder or rectal motion. Frame-level timestamps enabled time-resolved displacement analysis, with effective sampling frequencies of approximately 15 Hz in centers 1 and 3 and 5–6 Hz in centers 2 and 4. Cases from four participating centers formed the main analytic cohort for geometry processing, voxel-wise dose sampling, DVH recomputation, and multicenter geometry–dosimetry analyses. Although intrafraction motion-monitoring records were available in the source dataset, the primary analyses in the present study focused on associations between static anatomical geometry and planned RTDOSE-derived dosimetric endpoints. Institutional data from Ganzhou Cancer Hospital included planning CT and accompanying DICOM-RT objects (RTSTRUCT, RTPLAN, RTDOSE, and related files). This institutional dataset was used as an independent real-world clinical DICOM cohort to assess workflow applicability beyond the public multicenter dataset. It was not used for model development, formal hypothesis testing, parameter estimation, or external validation.

Cases were included when (1) a complete planning CT series and corresponding DICOM-RT data were available for the same patient (with RTSTRUCT required at minimum and RTPLAN/RTDOSE additionally required for dosimetric calculations); (2) DICOM reference relationships were resolvable so that RTSTRUCT could be unambiguously linked to the corresponding CT series for contour mapping; and (3) contours could be rasterized into three-dimensional structure masks, with RTDOSE metadata sufficient for dose-grid reconstruction and voxel-wise dose sampling. Cases were excluded when imaging or DICOM-RT files were incomplete, key metadata were unreadable, reference relationships were unresolved or invalid, CT–RTSTRUCT spatial consistency was insufficient and led to failed contour mapping or rasterization, or three-dimensional structure masks could not be generated during subsequent processing. In addition, the number of slices and slice thickness/spacing were recorded for each CT series to characterize potential resolution differences that might affect geometric measurements and the consistency of dose sampling.

The unit of analysis was defined at the patient–plan level. In the public dataset, each patient corresponded to a single treatment plan (i.e., one treatment course). Accordingly, unless otherwise specified, the terms “patient,” “plan,” and “treatment course” refer to the same analytic unit throughout the manuscript.

This study was approved by the Institutional Ethics Committee of Ganzhou Cancer Hospital (Approval No. [2026] KELUNSHEN No. 3). All patient data were anonymized prior to analysis in accordance with relevant ethical and data privacy regulations.

### 2.2 Radiotherapy planning and delivery

All radiotherapy plans were derived from routine clinical practice and analyzed as-is; no plan re-optimization or parameter adjustment was performed. Prescription dose and fractionation were extracted from RTPLAN and are summarized in **Table 1**. In the original dataset, 37 of 38 plans were delivered with a 5-fraction regimen, whereas one plan used single-fraction treatment. The single-fraction case corresponded to the prespecified low-dose target case (center3/Patient02; RTPLAN prescription dose 7.25 Gy) and was therefore not included in the final analytic cohort. Accordingly, all 37 cases in the final analytic cohort were treated with 5-fraction regimens. In the main analytic cohort, explicit RTPLAN target prescription-dose information was available for 33 of 37 plans, with a median prescribed dose of 36.25 Gy (range, 28.93–38.28 Gy). As a descriptive surrogate of prescription context, plan-level PTV D_95_ from the original plan is also summarized in **Table 1**.

**Table 1:** Overall cohort and plan-level characteristics (N = 38 plans; main analytic cohort n = 37).

Variable	Overall cohort (N = 38 plans)
Planning technique	
VMAT, n (%)	38 (100.0)
Fractionation	
1 fraction, n (%)	1 (2.6)
5 fractions, n (%)	37 (97.4)
Main analytic cohort: 5 fractions, n (%)	37 (100.0)
Contributing centers	
center1, n (%)	9 (23.7)
center2, n (%)	20 (52.6)
center3, n (%)	5 (13.2)
center4, n (%)	4 (10.5)
Prescription dose	
Prescription dose from explicit RTPLAN target dose reference (Gy), median (range)	36.25 (28.93–38.28)
Prescription-context surrogate	
PTV D_95_ (Gy), median (range)	36.24 (15.21–36.54)
Geometric metrics	
Bladder volume (cc), median (IQR; range)	695.2 (667.5; 192.1–2801.3)
Rectum volume (cc), median (IQR; range)	199.6 (107.6; 53.3–488.0)
Dosimetric metrics	
Bladder D_2cc_ (Gy), median (range)	36.41 (24.61–37.58)
Bladder D_mean_ (Gy), median	6.25
Rectum D_2cc_ (Gy), median (range)	35.47 (18.10–37.28)
Rectum D_mean_ (Gy), median	10.16

Plan-level summaries are shown for the overall cohort (N = 38 plans) unless otherwise specified. The main analytic cohort included 37 plans after excluding one low-dose target case due to abnormal target coverage; this excluded case was also the only single-fraction plan in the dataset. Accordingly, all plans in the main analytic cohort were delivered in 5 fractions. Prescription dose from explicit RTPLAN target dose reference was available in 33/37 main-cohort plans, with a median of 36.25 Gy (range, 28.93–38.28 Gy). Four center4 RTPLAN files contained fractionation information but did not include a structured target prescription-dose tag. Categorical data are presented as n (%). Continuous data are presented as median (IQR; range) or median (range). Units: cm³ (cc), Gy. D_2cc_: minimum dose to the most irradiated 2 cm³; D_mean_, mean dose; PTV D_95_, dose to 95% of the PTV.

Target volumes and organs at risk (OARs) were imported directly from RTSTRUCT, without re-contouring or manual correction. Target volumes were analyzed as clinically defined in the original plans. Because target-definition metadata were not fully standardized across centers, we did not retrospectively reclassify all cases into prostate-only versus prostate-plus-seminal-vesicle subgroups. Using a unified workflow, all contours were rasterized into 3D binary masks aligned to the planning CT grid. The original DICOM coordinate system, image orientation, and spatial resolution were preserved to ensure geometric consistency.

All DVH metrics were recomputed from RTDOSE using a standardized implementation (Section 2.5), aiming to minimize systematic differences introduced by vendor-specific DVH calculation and reporting.

### 2.3 Image processing and structure mask generation

All geometric computations and voxel-wise dose sampling were performed in the planning CT DICOM physical coordinate system. The planning CT was used solely as a spatial reference, and no image-intensity- or density-based analyses were conducted.

**Figure 1:**
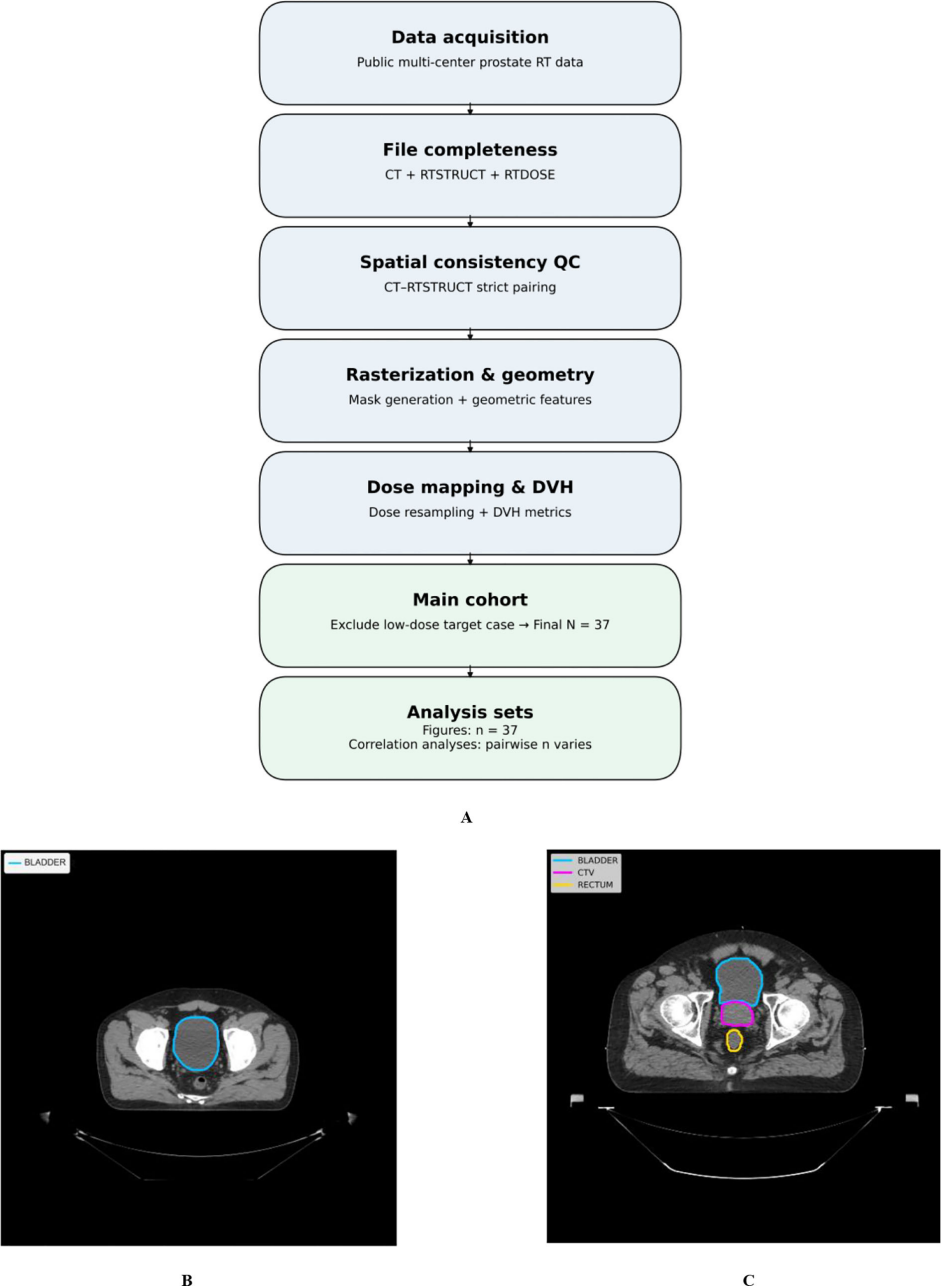
Study workflow and mask generation. **(A)** Overview of the data processing pipeline, including file completeness checks, CT–RTSTRUCT spatial consistency quality control, structure rasterization and geometric feature extraction, dose mapping, and DVH recalculation. One low-dose target case was excluded, yielding the main analytic cohort (n = 37). **(B)** Example of CT-based structure rasterization showing representative target and OAR masks in axial view. **(C)** Example of multi-ROI mask representation illustrating spatial relationships among representative structures in axial view. The displayed structures are identified in the figure legends (e.g., BLADDER, CTV, and RECTUM). RTSTRUCT, radiotherapy structure set; RTDOSE, radiotherapy dose; DVH, dose–volume histogram; PTV, planning target volume; CTV, clinical target volume.

Structure contours were obtained from RTSTRUCT. We parsed the ContourSequence and mapped contour points to the corresponding CT slices using DICOM spatial metadata (ImagePositionPatient, ImageOrientationPatient, and PixelSpacing). Contours were filled on each slice and stacked in slice order to form 3D binary masks. To ensure consistent processing across centers, we did not apply contour interpolation, spatial resampling, or morphological post-processing. The resulting masks were treated as voxel-wise representations of the clinically delineated structures on the native CT grid (**Figures 1B, C**).

To address between-center variability in structure naming, ROI labels were standardized using a predefined mapping table. Only ROIs that could be reliably consolidated were retained for downstream analyses (PTV, CTV, BLADDER, RECTUM, PENILE_BULB, and BODY). BODY was defined as the external patient contour as provided in RTSTRUCT over the available planning CT scan extent. We then applied ROI-level quality control, including non-emptiness checks, minimum voxel-count thresholds, and plausibility checks on spatial extent. Specifically, an ROI mask was required to be non-empty (voxels > 0), contain at least 10 voxels, have non-zero spatial extent in all three axes (bbox_x_mm > 0, bbox_y_mm > 0, bbox_z_mm > 0), and span at least two CT slices. In addition, scan-length coverage was audited using a scan-boundary touch flag (zmin = 0 or zmax = shape_z − 1) to identify potentially truncated structures. BODY boundary-touch findings were flagged and retained, whereas any boundary-touch finding in analysis-relevant ROIs (PTV, CTV, BLADDER, RECTUM, and PENILE_BULB) would have required manual review before inclusion. No analysis-relevant ROI showed scan-boundary touch in the audited dataset. ROIs failing QC were excluded only for that ROI, while the same case could remain eligible for other ROIs and subsequent analyses. At the ROI-row level, 22 structure-specific entries failed QC and were excluded from downstream analyses. These excluded entries comprised 8 BLADDER, 7 RECTUM, 6 PENILE_BULB, and 1 CTV ROI row; no PTV or BODY ROI rows were excluded at this stage. The corresponding patients remained eligible for analyses of other structures.

### 2.4 Geometric feature definition and computation

All geometric features were computed from the 3D binary ROI masks described in Section 2.3. Masks were defined on the native planning CT grid and therefore shared identical grid geometry (origin, spacing, and dimensions) in the same DICOM physical coordinate system. To maintain cross-center comparability and reduce processing-induced bias, masks were not resampled, interpolated, or morphologically post-processed.

#### 2.4.1 Volume

Let Ω_r_ denote the voxel set belonging to structure r, and |Ω_r_| its cardinality (voxel count). With voxel spacing Δx, Δy, Δz (mm), the structure volume is:


$$V_{r}=\left|{\text{Ω}}_{\text{r}}\right|\cdot \text{ΔxΔyΔz}$$

Volumes were converted to cm^3^ for statistical analysis (1cm^3^ = 1000mm^3^).

#### 2.4.2 Spatial extent

Spatial extent was computed using the axis-aligned bounding box (AABB). Let *x*_min_, *x*_max_, *y*_min_, *y*_max_, *z*_min_, *z*_max_ denote the minimum and maximum voxel indices of structure r along the x-, y-, and z-axes on the native CT grid. The physical extents (mm) were computed as:


$$E_{x}=\left(x_{max}-x_{min}+1\right)\Delta x,\;E_{y}=\left(y_{max}-y_{min}+1\right)\Delta y,\;E_{z}=\left(z_{max}-z_{min}+1\right)\Delta z$$

Given the higher sensitivity of pelvic dose gradients to superior–inferior (SI) geometry, the primary longitudinal extent was defined as:


$${\text{BBoxZ}}_{r}={\text{E}}_{z}$$

Together, V_r_ and BBoxZ_r_ formed the geometric feature set used for association analyses with DVH endpoints.

### 2.5 Dosimetric analysis and quality control

Dosimetric analyses were conducted using planned RTDOSE. The 3D physical dose grid was reconstructed from DICOM metadata (DoseGridScaling, ImagePositionPatient, PixelSpacing, and GridFrameOffsetVector) and expressed in absolute dose (Gy). Dose grids, planning CT, and ROI masks were handled within the same DICOM physical coordinate system. When mismatches existed between the dose grid and CT grid (resolution and/or voxel arrangement), only the dose grid was resampled to the CT grid using trilinear interpolation to enable consistent voxel-wise dose sampling on the native CT masks. ROI masks were kept on the native CT grid without interpolation to avoid systematic bias from boundary smoothing or volume drift.

For each ROI, voxel doses were sampled within the binary mask to form an ROI-specific dose distribution, and DVH metrics were recomputed using a unified pipeline to minimize variability attributable to vendor-specific planning systems or DVH implementations. Dose-at-volume metrics were derived by sorting voxel doses and accumulating voxel volumes without prespecified dose binning. Standard endpoints included D_mean_, D_max_, D_min_, and D_x_ (e.g., D_98_, D_95_, and D_2_), where D_x_ denotes the minimum dose received by at least x% of the ROI volume. For OARs, D_2cc_ was additionally computed as the dose to the hottest 2 cm^3^, implemented via voxel-volume accumulation.

Quality control was performed without *post hoc* numerical correction of atypical values. Instead, outlier cases were subjected to case-level auditing to differentiate geometric explanations (e.g., spatial separation from the high-dose region) from potential processing failures. A threshold of 0.5 Gy was used as a practical near-zero QC cut-off to identify bladder D_2cc_ values suggestive of a floor-effect geometry–dose regime rather than the usual non-zero continuous variation range. In total, 8 bladder cases met this criterion in the sensitivity dataset (8/35, 23.5%); 7 were exactly 0 Gy and 1 was greater than 0 Gy but still below 0.5 Gy. When D_2cc_ was used as an analysis endpoint, these QC-flagged near-zero cases were excluded from the primary continuous-correlation analysis, leaving 27 QC-filtered bladder cases for the main bladder D_2cc_ analysis. All other endpoints were analyzed using the main cohort after ROI-level QC, with the effective sample size (N) reported per analysis.

### 2.6 Statistical analysis

Statistical analyses were performed in the main cohort using two-sided tests, with statistical significance set at p< 0.05. Because the primary aim was to evaluate geometry–dosimetry associations, interpretation emphasized effect size and directionality rather than statistical significance alone. Continuous variables were summarized as median (interquartile range, IQR), and categorical variables as number (percentage). Non-parametric methods were used to reduce reliance on distributional assumptions.

Associations between geometric features and dosimetric endpoints were assessed using Spearman’s rank correlation coefficient (ρ) to quantify monotonic relationships. For each ROI–geometric metric–DVH endpoint pair, Spearman’s ρ and the corresponding two-sided p value were reported. Results were interpreted based on the magnitude of ρ, direction consistency across related endpoints, and clinical/biological plausibility.

Atypical dosimetric values (e.g., near-zero bladder D_2cc_ values, defined as bladder D_2cc_< 0.5 Gy) were not numerically adjusted. When bladder D_2cc_ was analyzed as a continuous endpoint in the primary Spearman analysis, QC-flagged near-zero cases were excluded from correlation testing because they represented a structural floor-effect regime rather than continuous variation within the usual non-zero dose range. To assess the impact of this endpoint-specific handling, we additionally performed a sensitivity analysis including the near-zero cases (defined as bladder D_2cc_< 0.5 Gy).

All analyses were conducted in Python (NumPy, SciPy, pandas). Because the single-fraction case corresponded to the prespecified low-dose target case and was excluded from the final analytic cohort, all cases in the main analytic cohort were treated with 5-fraction regimens. As a sensitivity analysis, selected key DVH endpoints were expressed as percentages of the explicit RTPLAN target prescription dose (%Rx) in the 33 of 37 main-cohort plans with available prescription-dose metadata, and the corresponding Spearman correlation analyses were repeated. Figures were generated using matplotlib.

## 3 Results

### 3.1 Cohort construction and analysis overview

We identified 38 eligible cases (one plan per patient) from a publicly available multicenter prostate radiotherapy dataset (**Figure 1**). After file-integrity screening and CT–RTSTRUCT spatial-consistency QC, no cases were excluded for missing data or spatial mismatch. The workflow and cohort selection are summarized in **Figure 1A**, with examples of structure rasterization and multi-ROI spatial relationships shown in **Figures 1B, C**.

A prespecified target-dose plausibility audit excluded one case (center3/Patient02) because of markedly low target prescription/coverage (“low-dose target” case). This case was excluded from all subsequent ROI-level dosimetric summaries. The excluded case was also the only single-fraction plan in the dataset. The final main cohort therefore included 37 cases for geometric feature extraction, DVH recomputation, and downstream association analyses, all treated with 5-fraction regimens.

All analyses used a unified processing pipeline without dose repair, *post hoc* normalization, or manual intervention. Because valid values may differ across ROI–endpoint pairs (e.g., due to ROI-level QC or endpoint-specific exclusions), the effective sample size varied by analysis; the actual N is reported in the corresponding tables/figures. Baseline cohort characteristics and key plan parameters are provided in **Table 1**. **Table 1** also summarizes treatment-context information relevant to interpretation of the absolute dosimetric endpoints, including fractionation scheme, prescribed dose derived from explicit RTPLAN target dose reference where available, and the distribution of plan-level PTV D_95_ values.

### 3.2 Quality-control audit of atypical dosimetric values

During dosimetric extraction, a total of 8 bladder cases exhibited near-zero bladder D_2cc_ values (bladder D_2cc_< 0.5 Gy) in the sensitivity dataset (8/35, 23.5%). Targeted geometry–dose consistency auditing indicated that these values were not caused by dose-grid reconstruction errors, DVH computation failures, or post-processing artifacts. Instead, they reflected a geometric configuration in which the bladder had little overlap with, or was spatially separated from, the target high-dose region. Of these 8 cases, 7 were exactly 0 Gy and 1 was non-zero but still below 0.5 Gy. All near-zero bladder D_2cc_ values (bladder D_2cc_< 0.5 Gy) occurred within the main analytic cohort and were distinct from the separately excluded “low-dose target” case (center3/Patient02). These cases were therefore interpreted as clinically plausible geometric extremes rather than merely statistical outliers.

In the flagged cases, the bladder lay predominantly outside the high-dose distribution. Accordingly, the hottest 2 cm³ within the bladder was not encompassed by the prescription or near–high-dose region, and voxel-wise sampling yielded near-zero dose values. This pattern therefore represents objective geometry–dose separation rather than a technical planning or computation error. We agree that these cases represent an important extreme of the geometry spectrum rather than meaningless outliers.

No abnormalities were identified in the original RTDOSE files, DoseGridScaling, spatial alignment, or reconstruction steps (e.g., unexpected rescaling, truncation, or post-processing distortion). Cases with near-zero bladder D_2cc_ values (bladder D_2cc_< 0.5 Gy) were flagged and handled according to a prespecified rule: when D_2cc_ was the endpoint, flagged cases were excluded from correlation analyses; otherwise, cases remained eligible for analyses not involving D_2cc_. Because these cases represent a distinct floor-effect regime, we additionally performed a sensitivity analysis including the near-zero cases (defined as bladder D_2cc_< 0.5 Gy). For bladder volume, the association direction was preserved and the correlation magnitude remained similar (main cohort ρ = 0.410; sensitivity ρ = 0.468). In contrast, for bladder longitudinal extent (bbox_z_mm), the correlation estimate changed substantially and the direction was not preserved (main cohort ρ = 0.509; sensitivity ρ = -0.126). These findings indicate that the near-zero cases can materially influence continuous correlation estimates for some geometric descriptors. The effective sample size (N) is reported for each analysis and figure.

### 3.3 Distribution of geometric features in key structures in the main cohort

**Figure 2:**
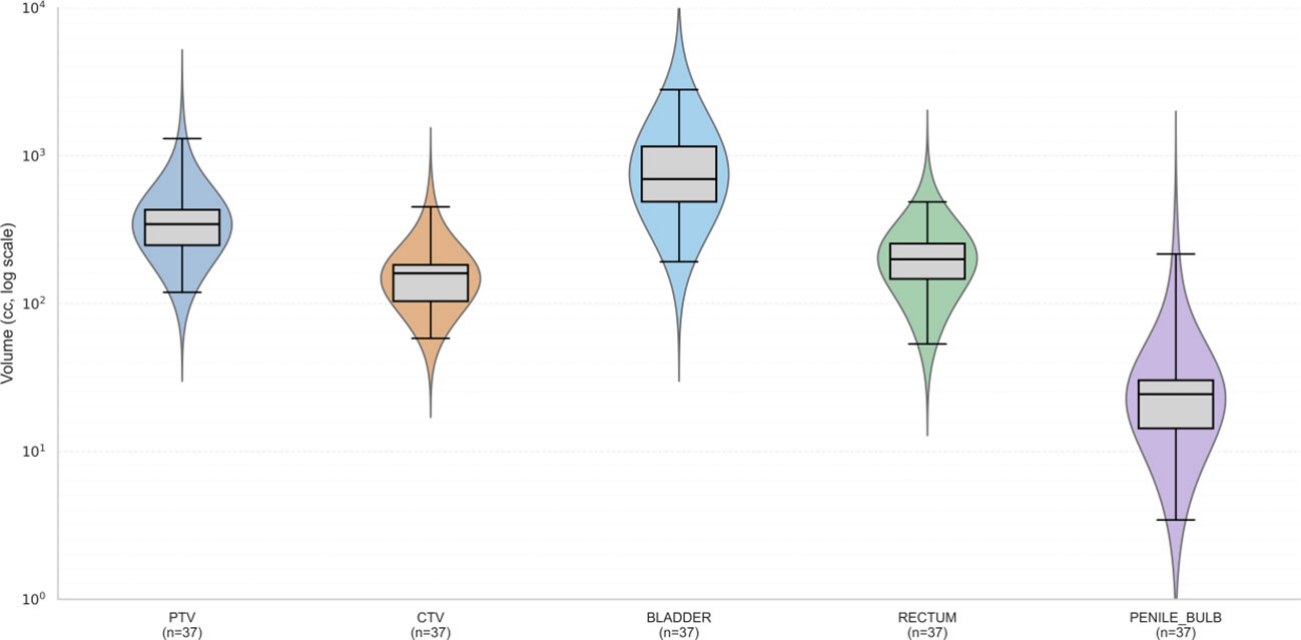
Volume of key anatomical structures. Volume distributions (log scale) of PTV, CTV, bladder, rectum, and penile bulb in the main cohort (n = 37) shown as violin plots with overlaid boxplots. Boxes indicate the interquartile range with the median, and whiskers denote the full data range.

Across the main cohort (n = 37), volumes of the evaluated structures, including bladder, varied markedly between patients (**Figure 2**); BODY is omitted for clarity.

Bladder and rectal volumes showed substantial inter-patient variability, whereas PTV and CTV volumes clustered more narrowly with smaller interquartile ranges, consistent with relatively stable target delineations in this multicenter dataset. The penile bulb showed the greatest dispersion and a skewed distribution.

These patterns underscore structure-dependent geometric variability relevant to downstream dosimetric association results.

### 3.4 Intrafraction motion context of the public multicenter cohort

**Figure 3:**
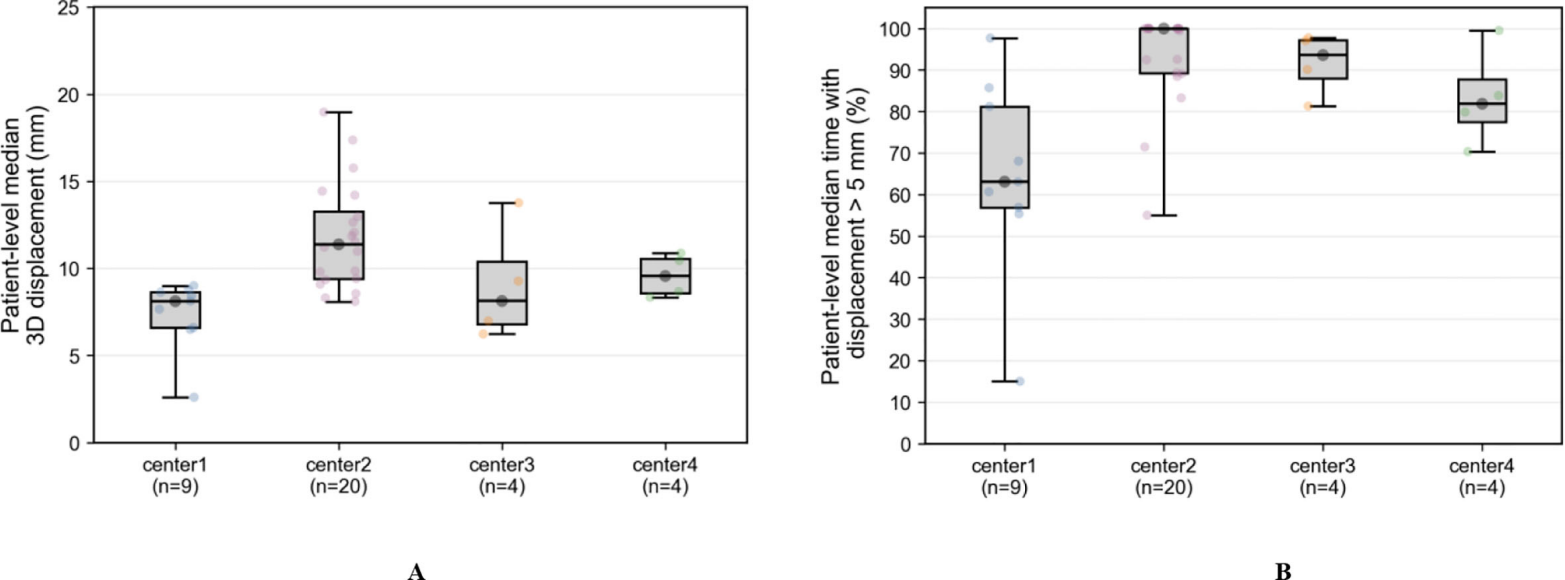
Intrafraction motion context by center. **(A)** Patient-level median 3D displacement (disp3D) by center. **(B)** Patient-level motion burden, defined as the percentage of treatment time with displacement ≥ 5 mm, by center. Boxplots show the median and interquartile range, with whiskers indicating the full data range. Points represent individual patients. These summaries are shown to characterize the motion-monitoring context of the public dataset and are not intended as direct motion–dose association analyses.

Intrafraction motion-monitoring records available in the public dataset were summarized at the patient level to describe the image-guided treatment context of the source cohort (**Figure 3**). Substantial inter-patient variability was observed in all centers for both displacement magnitude and motion burden.

For displacement magnitude, disp3D_median showed the following center-specific statistics: center1 (n = 9): median 8.11 mm, IQR 6.60–8.63 mm; center2 (n = 20): median 11.38 mm, IQR 9.39–13.28 mm; center3 (n = 4): median 8.12 mm, IQR 6.79–10.39 mm; center4 (n = 4): median 9.56 mm, IQR 8.58–10.56 mm (**Figure 3A**).

Between-center differences were more evident for motion burden (time>5mm% median and IQR: center1: 63.09%, 56.92–81.22%; center2: 100.00%, 89.29–100.00%; center3: 93.59%, 87.92–97.23%; center4: 81.86%, 77.48–87.77%; **Figure 3B**). These summaries are presented to characterize the treatment context of the source dataset and are not intended as direct motion–dose association analyses.

### 3.5 Associations between geometric features and DVH endpoints

After QC flagging of atypical values (near-zero bladder D_2cc_ values, defined as bladder D_2cc_< 0.5 Gy), we assessed associations between geometric features and DVH endpoints in the main cohort (n = 37). All correlations reported in **Table 2** were calculated in the main cohort (n = 37). Overall, the most consistent correlation patterns were observed for OARs, particularly bladder-related endpoints.

**Table 2:** Spearman correlations between geometric features and DVH metrics (n = 37).

ROI	Geometric feature	DVH metric	Spearman’s ρ	P value
Bladder	Volume (cc)	D_95_ (Gy)	−0.78	<0.001
	Volume (cc)	D_mean_ (Gy)	−0.76	<0.001
	Longitudinal extent (bbox_z, mm)	D_95_ (Gy)	−0.72	0.002
Rectum	Volume (cc)	D_max_ (Gy)	0.43	0.010
	Volume (cc)	D_2cc_ (Gy)	0.41	0.014
	Volume (cc)	D_mean_ (Gy)	0.40	0.019
	Volume (cc)	D_2_ (Gy)	0.39	0.019
Body	Volume (cc)	D_mean_ (Gy)	−0.77	<0.001
	Volume (cc)	D_2_ (Gy)	−0.71	0.002
Penile bulb	Volume (cc)	D_min_ (Gy)	−0.69	0.003
PTV	Longitudinal extent (bbox_z, mm)	D_max_ (Gy)	0.60	0.015
CTV	Volume (cc)	D_max_ (Gy)	0.43	0.097

Spearman’s rank correlation coefficient (ρ) was computed using two-sided tests. All correlations in this table were calculated in the main cohort (n = 37). D_x_ indicates the minimum dose received by at least x% of the structure volume. D_2cc_ indicates the dose to the hottest 2 cm³ of an organ. Cases with bladder D_2cc_< 0.5 Gy identified by quality control were excluded from the primary D_2cc_-related continuous-correlation analyses.

Bladder volume (volume_cc) showed a strong inverse association with the representative near-minimum dose endpoint D_95_ (Spearman’s ρ = −0.78, p< 0.001) and was also inversely associated with bladder mean dose (D_mean_) (**Table 2**). Bladder longitudinal extent also showed a negative correlation with bladder D_95_ (**Table 2**).

Rectum volume also showed positive correlations with several rectal DVH metrics, including D_max_, D_2cc_, D_mean_, and D_2_, whereas rectum longitudinal extent showed weaker and less consistent associations (**Table 2**).

For completeness, BODY volume was also inversely correlated with BODY D_mean_ and D_2_, and penile bulb volume was moderately inversely correlated with penile bulb D_min_ (**Table 2**). However, BODY should be interpreted cautiously because it represents the external contour over the available scan extent and may therefore be influenced by variation in CT scan length.

In contrast, geometric–dosimetric associations were weaker for targets. PTV longitudinal extent was moderately positively correlated with PTV D_max_, whereas the association between CTV volume and CTV D_max_ did not reach statistical significance (**Table 2**). These target-related entries were retained in **Table 2** as representative exploratory associations selected for interpretability, rather than to imply that only these specific target features were considered.

To assess the impact of prescription-dose variation on cross-patient comparability, we additionally performed a prescription-normalized sensitivity analysis in the subset of cases with explicit RTPLAN target prescription-dose information available. The principal bladder-related associations remained directionally consistent after normalization: bladder volume remained inversely associated with bladder D_95_ (%Rx) (ρ = −0.76, p< 0.001) and bladder D_mean_ (%Rx) (ρ = −0.76, p< 0.001), and bladder longitudinal extent remained inversely associated with bladder D_95_ (%Rx) (ρ = −0.46, p = 0.007). For rectum volume versus rectum D_2cc_, the positive association direction was preserved after normalization (ρ = 0.36, p = 0.076), although the association no longer reached conventional statistical significance in the reduced prescription-available subset (**Table 3**).

**Table 3:** Prescription-normalized sensitivity analysis of selected DVH associations.

ROI	Geometric feature	DVH metric	Spearman’s ρ	P value
Bladder	Volume (cc)	D_95_ (%Rx)	−0.76	<0.001
	Volume (cc)	D_mean_ (%Rx)	−0.76	<0.001
	Longitudinal extent (bbox_z, mm)	D_95_ (%Rx)	−0.46	0.007
Rectum	Volume (cc)	D_2cc_ (%Rx)	0.36	0.076
		D_mean_ (%Rx)	0.13	0.469

Spearman’s rank correlation coefficient (ρ) was computed using two-sided tests. Prescription-normalized sensitivity analyses were restricted to cases with explicit RTPLAN target prescription-dose information available (n = 33 unless otherwise limited by endpoint-specific exclusion criteria). %Rx indicates the corresponding DVH metric divided by prescription dose and multiplied by 100. For rectum volume versus rectum D2cc (%Rx), the effective sample size was n = 25.

### 3.6 Workflow applicability in an independent institutional DICOM cohort

We additionally applied the same processing workflow to an independent institutional cohort of prostate radiotherapy cases (n = 8) from Ganzhou Cancer Hospital. This cohort was not included in the primary multicenter analysis and was not used for model development, formal hypothesis testing, parameter estimation, or external validation. Instead, it was used to assess whether the workflow could be implemented in an independent real-world clinical DICOM setting. Institutional cases were processed with the same pipeline as the main cohort, including ROI rasterization, geometric feature computation, and DVH recomputation. The workflow was successfully executed in the institutional cohort, supporting its applicability beyond the public multicenter dataset.

## 4 Discussion

### 4.1 Principal findings

In this study, using a publicly available multicenter prostate radiotherapy dataset (13), we evaluated how static anatomical geometry was associated with planned dosimetric endpoints within a standardized pipeline for geometric feature extraction, dose mapping, and quality control (QC) (14, 15). Under contemporary planning and PTV-margin practices, OAR dose variability showed robust and directionally consistent associations with inter-patient static anatomy, most notably bladder volume and longitudinal extent (16, 17). A prescription-normalized sensitivity analysis in the 33 main-cohort plans with explicit RTPLAN target prescription-dose information showed directionally consistent bladder-related associations, suggesting that modest between-patient prescription-dose variation did not drive the main interpretation. From a clinical workflow perspective, geometry-derived information is not intended to replace standard inverse planning, physician review, or routine IGRT. Rather, it may serve as an early planning-support signal. Because these geometric features can be computed directly from the planning CT and RTSTRUCT before or during plan generation, they may help identify cases in which less favorable target–OAR geometry is more likely to be associated with less favorable OAR dose trade-offs. In practice, this information could support closer review of OAR sparing, reinforcement of bladder/rectal preparation protocols, and consideration of re-simulation or re-planning when anatomy is judged suboptimal. Compared with more complex approaches such as OVH/DTH-based modeling or deformable delivered-dose accumulation (18–20), the present framework relies on simple CT-derived geometric descriptors that can be computed directly from standard planning CT and RTSTRUCT data within a unified multicenter QC workflow. Its role is therefore not to replace detailed geometric–dosimetric modeling or delivered-dose reconstruction, but to provide a low-complexity signal that may be most usefully implemented as an early planning-support or QC tool.

### 4.2 Clinical interpretation of geometry–dose associations

For OARs, both bladder volume and longitudinal extent were inversely associated with the representative near-minimum dose endpoint D_95_. This pattern is not unexpected and is intuitively consistent with pelvic geometry, because a larger bladder—particularly one with greater cranio–caudal extent—would be expected to place a greater proportion of bladder tissue farther from the high-dose region (21). Compared with volume alone, longitudinal extent captures spatial occupancy along the cranio–caudal axis that volume cannot reflect, providing a complementary quantitative description of geometric coupling between OARs and high-dose regions (22, 23). In contrast, geometric features of PTV/CTV showed weaker associations with target-coverage metrics, which may reflect improved coverage robustness under modern optimization and margin strategies, with reduced sensitivity to inter-patient anatomical variation (24). This pattern was not unexpected, because contemporary inverse planning and margin-based design intentionally constrain target coverage variability across patients. We therefore interpret these target-related metrics as secondary exploratory endpoints rather than the principal clinical focus of the present analysis.

### 4.3 Interpretation of atypical values and workflow applicability

Near-zero bladder D_2cc_ values observed in a subset of cases were not consistent with obvious errors in dose reconstruction or DVH computation. Instead, they reflected a geometric configuration with minimal or no overlap between the bladder and the high-dose region. In the present study, 8 bladder cases met the near-zero criterion of D_2cc_< 0.5 Gy; 7 were exactly 0 Gy and 1 was greater than 0 Gy but still below 0.5 Gy. Their exclusion from the primary Spearman analysis was intended to separate a structural floor-effect regime from the continuous monotonic variation observed across the remaining non-zero cases, rather than to dismiss them as meaningless outliers. These cases are better interpreted as clinically plausible geometric extremes rather than merely statistical outliers. The sensitivity analysis showed that this handling had feature-dependent consequences: the association with bladder volume remained directionally stable, whereas the association with longitudinal extent was not robust to inclusion of the near-zero cases. Accordingly, interpretation of bladder D_2cc_ associations, especially for longitudinal extent, should be made with caution. This interpretation is consistent with established approaches that explain DVH variation using geometric relationships between targets and OARs, including overlap- and distance-based descriptors (18). Therefore, QC review of atypical cases was used primarily to support result interpretation, rather than as a central study endpoint (25).

We also showed that the same workflow could be applied to an independent institutional DICOM cohort, supporting its practical applicability beyond the public multicenter dataset. This should not be interpreted as formal external validation (26).

### 4.4 Limitations and future directions

Several limitations merit consideration. The sample size was modest. The present study focused on geometry–dosimetry associations based on planned RTDOSE-derived static DVH metrics and did not include delivered-dose reconstruction, deformable dose accumulation, or explicit modeling of organ deformation or interfraction anatomical change. Moreover, BODY was defined as the external contour over the available CT scan extent. Accordingly, BODY-based volume and dosimetric metrics may have been influenced by variation in scan length and should be interpreted as descriptive rather than organ-specific geometric findings. Prior work indicates that interfraction anatomical variation can materially affect delivered dose, and that dose accumulation in the presence of deformation generally requires deformable image registration–based modeling (19, 20). In addition, the single-fraction case in the original dataset corresponded to the prespecified low-dose target case and was excluded from the final analytic cohort; accordingly, all cases in the final analytic cohort were treated with 5-fraction regimens, and the present findings should be interpreted in that treatment context. Explicit RTPLAN target prescription-dose metadata were unavailable in 4 of 37 main-cohort plans; therefore, prescription-normalized sensitivity analyses could only be performed in the remaining 33 plans, although the principal bladder-related associations remained directionally consistent in that subset. Finally, because target-definition metadata were not fully standardized across centers, we did not retrospectively separate all cases into prostate-only versus prostate-plus-seminal-vesicle subgroups.

In future studies, larger multi-institutional datasets, combined with deformable modeling and time-resolved delivered-dose reconstruction, will be needed to further validate geometry–dose relationships under more realistic delivery conditions (19, 20). Such studies could also evaluate these geometric features for plan-quality auditing and detection of atypical dosimetric patterns.

## 5 Conclusions

Using a unified geometry–dosimetry framework, we found that inter-patient variability in static anatomical geometry was associated with planned OAR dosimetric endpoints under a planned-dose (RTDOSE-based), static-DVH evaluation. Bladder volume and longitudinal extent showed stable, directionally concordant associations with key dosimetric endpoints, and prescription-normalized sensitivity analyses supported the robustness of the principal bladder-related findings. These findings support the value of anatomical-consistency control and geometry-informed planning review in prostate radiotherapy.

## Data Availability

The publicly available data analyzed in this study were obtained from the TROG 15.01 SPARK clinical trial dataset (NCT02397317). The institutional clinical data from Ganzhou Cancer Hospital are not publicly available because of ethical and patient privacy restrictions and were approved for use only within the scope of this study. Requests for access to the institutional data should be directed to Weixiang Lin (609591064@qq.com) and will be considered subject to approval by the relevant ethics committee and institutional data-sharing requirements.
